# Prognostic assessment in patients with newly diagnosed small cell lung cancer brain metastases: results from a real-life cohort

**DOI:** 10.1007/s11060-019-03269-x

**Published:** 2019-08-27

**Authors:** Ariane Steindl, Franziska Schlieter, Thomas Klikovits, Elena Leber, Brigitte Gatterbauer, Josa M. Frischer, Karin Dieckmann, Georg Widhalm, Sabine Zöchbauer-Müller, Mir Ali Reza Hoda, Matthias Preusser, Anna S. Berghoff

**Affiliations:** 1 Division of Oncology, Department of Medicine I, Medical University of Vienna, Vienna, Austria; 2 Comprehensive Cancer Center, Medical University of Vienna, Vienna, Austria; 3 Division of Thoracic Surgery, Medical University of Vienna, Vienna, Austria; 4 Department of Neurosurgery, Medical University of Vienna, Vienna, Austria; 5 Department of Radiotherapy, Medical University of Vienna, Vienna, Austria

**Keywords:** SCLC brain metastases, Prognostic factors, Prognostic scores, Neurological symptom, Synchronous diagnosis of BM and primary tumor

## Abstract

**Purposes:**

Brain metastases (BM) are a frequent complication in small cell lung cancer (SCLC), resulting in a reduced survival prognosis. Precise prognostic assessment is an important foundation for treatment decisions and clinical trial planning.

**Methods:**

Patients with newly diagnosed SCLC BM were identified from the Vienna Brain Metastasis Registry and evaluated concerning prognostic factors.

**Results:**

489 patients (male 62.2%, female 37.8%; median age 61 years) were included. Neurological symptoms were present in 297/489 (60.7%) patients. A- to oligosymptomatic patients (5 vs. 9 months, p = 0.030) as well as patients with synchronous diagnosis of BM and primary tumor (5 vs. 9 months, p = 0.008) presented with improved overall survival (OS) prognosis. RPA (HR 1.66; 95% CI 1.44–1.91; p < *0.001*), GPA (HR 1.65; p < *0.001*), DS-GPA (HR 1.60; p < *0.001*) and LabBM score (HR 1.69; p < *0.001*) were statistically significantly associated with OS. In multivariate analysis, DS-GPA (HR 1.59; p < 0.001), neurological deficits (HR 1.26; p = 0.021) and LabBM score (HR 1.57; p < 0.001) presented with statistical independent association with OS.

**Conclusion:**

A- to oligosymptomatic BM as well as synchronous diagnosis of SCLC and BM were associated with improved OS. Established prognostic scores could be validated in this large SCLC BM real-life cohort.

**Electronic supplementary material:**

The online version of this article (10.1007/s11060-019-03269-x) contains supplementary material, which is available to authorized users.

## Background

Brain metastases (BM) are the most common tumors of the central nervous system (CNS) and associated with a decline of survival time [1]. Small cell lung cancer patients have a particularly high propensity to develop BM as 10–20% of patients present with BM already at diagnosis of the primary tumor and up to 50% experience symptomatic BM during their course of disease [2, 3]. Treatment possibilities for symptomatic BM are limited and mainly based on local approaches including neurosurgical resection, stereotactic radiosurgery (SRS) and whole-brain radiation therapy (WBRT) [4]. Chemotherapy has only limited value in BM treatment of SCLC patients, although some small studies support a clinically meaningful intracranial activity [5, 6]. Prophylactic cranial irradiation (PCI), has been investigated extensively to prevent the occurrence of symptomatic BM, however is discussed controversially due to the lack of survival improvement in the light of severe, quality-of-life impairing side effects [7].

Precise survival prognosis estimation is an essential foundation for therapeutic decisions in patients suffering from SCLC BM. In this highly palliative setting with survival times ranging from few days to months, therapeutic approaches have to carefully consider a personalized benefit-risk ratio [8]. Standard prognostic scores such as the graded prognostic assessment (GPA), recursive partitioning analysis (RPA) and the more validated Diagnostic-Specific Graded Prognostic Assessments (DS-GPA) are based on clinical characteristics like patient age, Karnofsky Performance Scale (KPS), status of the extracranial disease, number of BM and primary tumor type [8–10]. The recently introduced LabBM score includes standard laboratory values to provide a more accurate survival estimation [11]. In the present study we aimed to investigate clinical prognostic factors, established prognostic scores as well as the newly introduced LabBM score in a large, real life cohort of SCLC BM patients treated at the Medical University of Vienna over a time period of more than 20 years.

## Methods

### Patients

Patient treated for SCLC BM at the Medical University of Vienna between 1990 and 2018 were identified from the Vienna Brain Metastasis Registry [12]. Clinical characteristics including course of disease, applied therapies and survival times were retrospectively evaluated by chart review. All patients were treated according to best clinical practice based on the discussion in a multidisciplinary tumor board.

SCLC were divided into limited and extensive disease according to published clinical practice guidelines of the European Society of Medical Oncology (ESMO) [13]. Prognostic scores including RPA, GPA and DS-GPA were calculated based on clinical characteristics as published previously [7, 9, 11]. In short, age, number of BM, status of the extracranial disease and KPS were evaluated and combined in the DS-GPA class for lung cancer [8]. The LabBM score was calculated as previously published based on serum heamoglobin concentration, platelet counts, serum albumin concentration, serum lactate dehydrogenase concentration and serum c-reactive protein concentration [11].

Synchronous diagnosis was defined as diagnosis of primary tumor and BM/extracranial metastasis within 30 days. Neurological symptoms were defined according to the written reports as either neurological deficits (any of the following: motor disorders, hypoesthesia, cranial nerve disfunction, cognitive impairment, vertigo and organic brain disorder), or signs of increased intracranial pressure (any of the following: headache, nausea, emesis) or epileptic seizures (focal or generalized seizures). If any of these neurological symptoms was presented the patient was categorized as symptomatic. If none of the listed neurological symptoms was present the patient was categorized as a- to oligosymptomatic. High symptomatic burden was defined as the presence of two or more neurological symptoms at BM diagnosis.

End of life disease status was evaluated based on the last available complete re-staging including extracranial as well as intracranial disease status within the last 60 days of life. Death due to intracranial progression was indicated in the absence of extracranial progression but clear increase in intracranial tumor burden. In contrast, death due to extracranial progression was defined in absence of intracranial progression, but increase in extracranial tumor burden in the last re-staging. Patient data were collected in a password secured database and handled anonymously. The study was approved by the ethics committee of the Medical University of Vienna (vote 078/2004).

### Statistical analysis

Overall survival time (OS) from primary tumor diagnosis was defined as time from initial histological diagnosis of the primary tumor to death or last follow-up. OS from BM diagnosis was defined as the time from initial radiological diagnosis of BM to death or last follow-up. Brain metastatic free survival was defined as time from diagnosis of primary tumor to radiological diagnosis of BM. Patients with synchronous diagnosis (within 30 days) of primary tumor and BM were excluded from analysis investigating the brain metastatic free survival period.

Chi square test was applied to analyze differences between two dichotomous variables. Kaplan Meier product limit methods were used for survival estimations and the log rank test was applied to analyze survival differences between groups. Parameters with statistically significant association with survival prognosis in the univariate analysis were included in multivariate analysis with the established prognostic scores using the Cox proportional hazard model. A two-sided p-value of < 0.05 was considered to indicate statistical significance. Due to the exploratory and hypothesis-generating design of the present study, no adjustment for multiple testing was applied [14].

## Results

### Patients characteristics

489 patients (male 304/489 (62.2%); female 185/489 (37.8%)) with newly diagnosed SCLC BM were available for further analysis. Median age at diagnosis of SCLC was 61 years (range 38–88 years). Table 1 and Supplementary Table 1 lists further patient’s and clinical characteristics.

**Table 1:** Clinical and patient’s characteristics

Characteristics at BM diagnosis	Entire population (n = 489)	
	n	%
Gender		
Male	304	62.2
Female	185	37.8
Median age at BM diagnosis	61(38–89)	
Median time from diagnosis of SCLC to BM development (range)	5 (0–102)	
Status of SCLC at BM diagnosis		
Synchronous diagnosis of extracranial metastases and BM	198	40.5%
Stable disease	112	22.9%
Progressive disease	102	20.9%
Complete response	3	0.6%
Partial response	61	12.5%
No evidence of extracranial disease after surgical resection of SCLC	13	2.7%
Synchronous diagnosis of extracranial metastases and BM		
Yes	156	31.9%
No	333	68.1%
Median Karnofsky Performance Score	80 (10–100)	
Neurological symptoms		
Present	340	69.5%
Absent (diagnosis during screening)	149	30.5%
Neurological deficits		
Present	297	60.7%
Absent	192	39.3%
Signs of increased intracranial pressure		
Present	122	24.9%
Absent	367	75.1%
Seizures		
Present	66	13.5%
Absent	423	86.5%
Number of neurological signs at BM diagnosis		
1	201	41.1%
≥ 2	139	28.4%
Characteristics after BM diagnosis		
First line treatment of BM		
SRS	212	43.4%
Chemotherapy	8	1.6%
Surgery	75	15.3%
WBRT	176	36.0%
Best supportive Care	18	3.7%
Systemic progression after first line BM treatment		
Yes	248	50.7%
No	241	49.3%
Median time from first BM treatment to systemic progression in months (range)	3 (0–91)	
Intracranial progression after first line BM treatment		
Yes	204	41.7%
No	285	58.3%
Median time from first BM treatment to intracranial progression in months (range)	7 (0–77)	
Status at last follow-up		
Deceased	444	90.8%
Alive	45	9.2%
Median overall survival from diagnosis of BM (range)	6 (0–264)	

*BM* brain metastasis, *SCLC* small cell lung cancer, *PCI* Prophylactic cranial irradiation, *SRS* Stereotactic radiosurgery, *WBRT* Whole brain radiation therapy

### Clinical characteristics associated with time to SCLC BM development

198/489 (40.5%) patients presented with synchronous diagnosis of BM and primary tumor and in consequence did not receive any tumor related treatment before the diagnosis of BM. Median BM free survival of the 291/489 (59.5%) patients with subsequent BM diagnosis was 10 months (range 3–102).

**Fig. 1:**
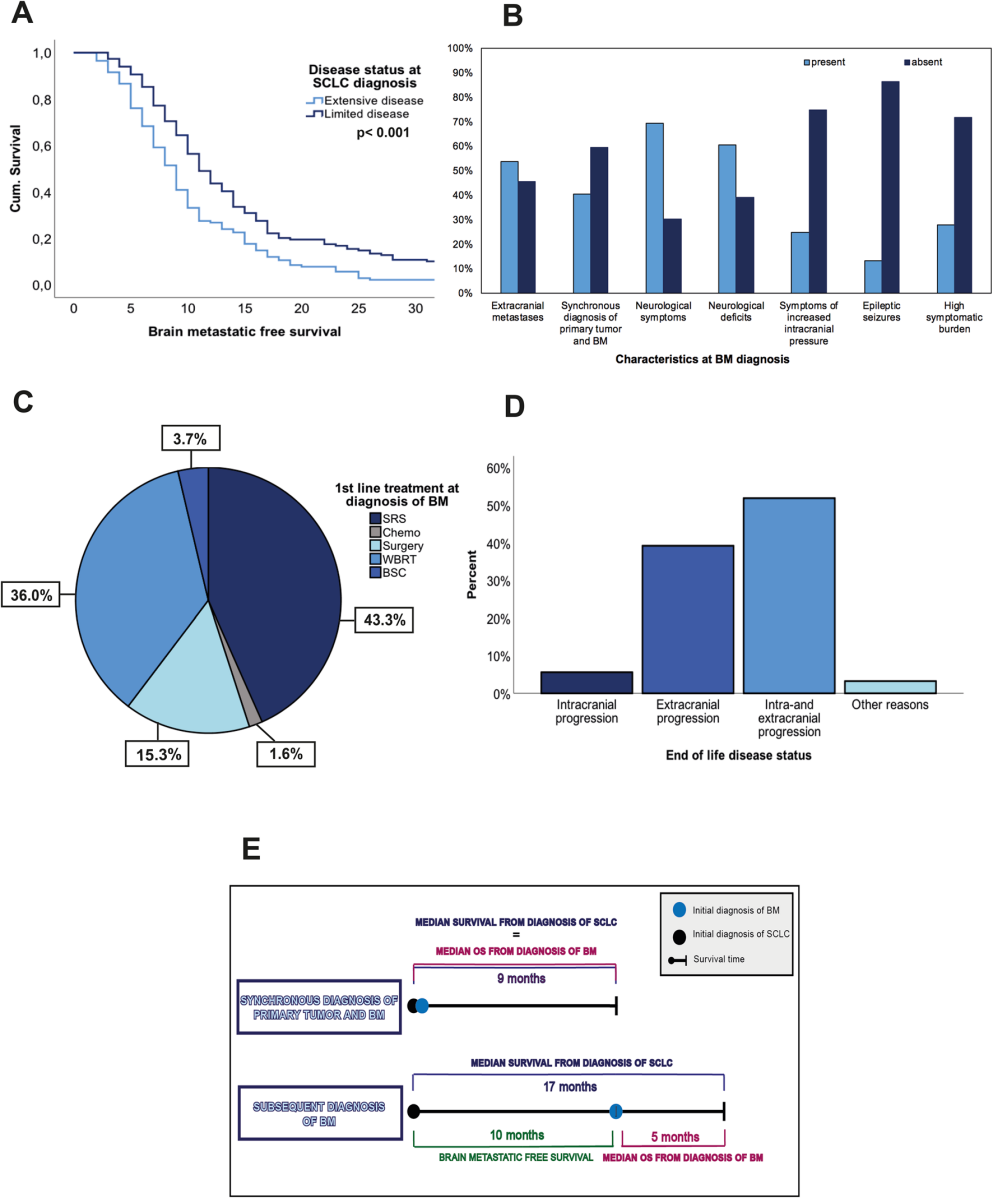
Clinical characteristics during the course of SCLC disease including (**a**) brain metastatic free survival in extended compared to limited disease SCLC according disease status at SCLC diagnosis (**b**) patient’s characteristics at BM diagnosis (**c**) first line treatment at diagnosis of BM (**d**) end of life disease status (**e**) overall survival according to synchronous compared to subsequent diagnosis of BM

158/489 (32.3%) patients diagnosed with limited SCLC showed a median brain metastatic free survival of 11 months (range 3–102) while 331/489 (67.7%) patients with extensive SCLC a median brain metastatic free survival of 9 months (range 2–42; p < 0.001; log-rank test; Fig. 1a).

### Clinical characteristics at SCLC BM diagnosis

Median age at BM diagnosis was 61 years (range 38–89 years). 149/489 (30.5%) patients were diagnosed with BM during routine screening or staging procedures in absence of BM specific symptoms. In the time period from 1990–2000, less patients were diagnosed in a- to oligosymptomatic stage due to screening (43/178; 24.2%) compared to patients diagnosed after 2000 (106/311; 34.1%; p = 0.022; chi-square test). Overall, 340/489 (69.5%) patients presented with at least one neurological symptom at BM diagnosis. In more detail, 297/489 (60.7%) patients suffered from neurological deficits, 122/489 (24.9%) patients from signs of increased intracranial pressure and 66/489 (13.5%) patients presented with seizures. 139/489 (28.4%) patients presented with highly symptomatic BM disease at diagnosis as 2 or more neurological symptoms were presented (Fig. 1b; Table 1).

Of 198/489 (40.5%) patients with synchronous diagnosis of SCLC and BM, 59/198 (29.8%) presented with a- to oligosymptomatic intracranial disease, while 139/198 (70.2%) patients presented with neurological symptoms (p = 0.790; chi-square test). Of 291/489 (59.5%) patients with subsequent BM diagnosis, 30.9% (90/291) were diagnosed with a- to oligosymptomatic intracranial disease, while 69.1% (201/291) of the patients suffered from neurological symptoms. (p = 0.13; chi-square test; Fig. 1c).

Table 1 and Supplementary Table 1 lists further patient’s and clinical characteristics at BM diagnosis.

### Clinical course after diagnosis of SCLC BM

212/489 (43.4%) patients were treated with SRS as the first line treatment. Of these, 62.3% (132/212) presented with neurological symptoms at BM and in 50% (106/212) SRS was performed to singular BM, in 37.3% (79/212) to 2–3 BM and in 12.7% (27/212) to ≥ 4 BM (Supplementary Table 1). In 42/212 (19.8%) patients treated with SRS, additional adjuvant WBRT was applied after SRS (Supplementary Table 1).

176/489 (36.0%) patients were treated with WBRT as the first line treatment approach. 58% (102/176) of these patients were diagnosed with ≥ 4 BM and 123/176 (69.9%) WBRT treated patients presented in a symptomatic status at BM diagnosis. (Supplementary Table 1).

Surgical resection as initial therapy of BM was performed in 75/489 (15.3%) patients (Fig. 1c; Table 1). Of these, 92.0% (69/75) suffered from neurological symptoms at BM diagnosis and 65.3% (49/75) were diagnosed with singular BM.

248/489 (50.7%) patients experienced systemic progression after first line BM treatment. Median time to extracranial progression was 3 months (range 0–91 months). 204/489 (41.7%) patients presented with intracranial progression with a median time to intracranial progression of 7 months (range 0–77 months). No difference in the time to intracranial progression (p > 0.05; log-rank test) or the development of intracranial progression (p > 0.05; chi-square test) according to applied first line BM treatment approach was evident.

The cause of death based on a complete re-staging within 60 days before death could be evaluated in 339/444 (76.04%) patients (Fig. 1d; Table 1). In 19/339 (5.6%) cases isolated intracranial progression in the absence of extracranial progression was the leading cause of death. 133/339 (39.2%) SCLC BM patients presented with progressive systemic disease in the absence of intracranial progression. In 176/339 (52.0%) combined intra- and extracranial progression was observed in the last re-staging examination before death. 11/339 (3.2%) patients died due to non-cancer related cause.

### Survival time according to clinical characteristics at SCLC BM diagnosis

Median overall survival from BM diagnosis was 6 months (range 0–264) in the entire cohort. Median survival from SCLC diagnosis was 9 months in patients with synchronous BM diagnosis and 17 months in patients with subsequent BM diagnosis (p < 0.001; log-rank test; Fig. 1e; Table 2). However, median OS from diagnosis of BM was longer in patients with synchronous diagnosis of primary tumor and BM (9 months) compared to patients with subsequent BM diagnosis after primary SCLC diagnosis (5 months; p = 0.008; log-rank test; Fig. 1e; Table 2).

**Table 2:** Clinical characteristics associated with OS

Characteristics	Entire population (n = 489)	
	OS (median) in months	p-value
Year of diagnosis		
> 2000	7	0.056
≤ 2000	6	
Synchronous diagnosis of SCLC and BM		0.008
Yes	9	
No	5	
Neurological symptoms		0.022
Yes	5	
No	7	
Neurological deficits		0.001
Yes	5	
No	8	
Signs of increased intracranial pressure		0.692
Yes	6	
No	6	
Seizures		0.828
Yes	5	
No	7	
Symptomatic burden		0.373
High	5	
Low	6	
First line treatment approach		< 0.001
WBRT	5	
SRS	7	
Neurosurgical resection	11	

*BM* brain metastasis, *OS* overall survival, *SCLC* small cell lung cancer, *SRS* Stereotactic radiosurgery, *WBRT* Whole brain radiation therapy

**Fig. 2:**
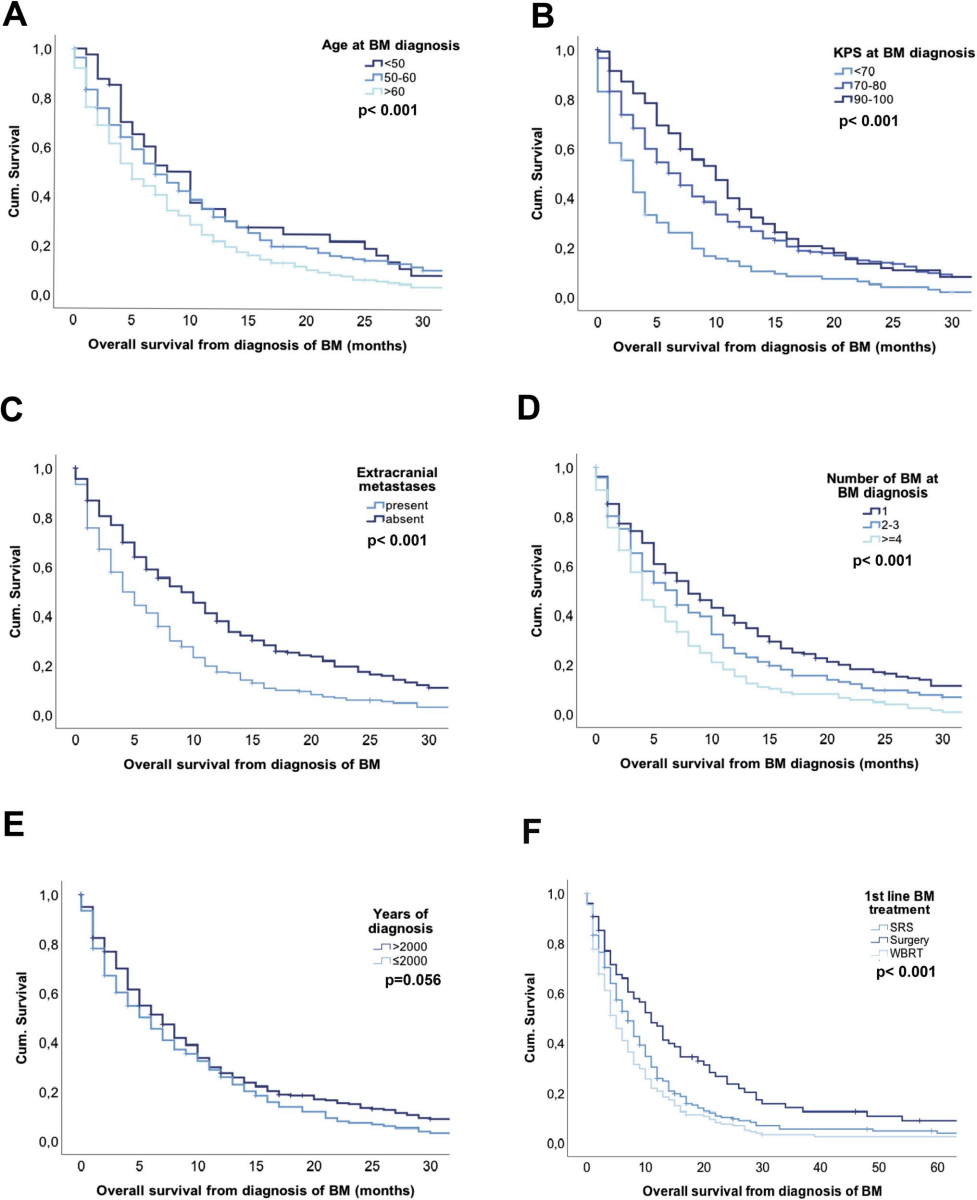
Overall survival according to (**a**) age at BM diagnosis (**b**) KPS at BM diagnosis (**c**) presence of extracranial disease at BM diagnosis (**d**) number of BM at diagnosis (**e**) year of BM diagnosis (**f**) first-line BM treatment approaches

Single parameters included in the RPA, GPA or DS-GPA including age, KPS, status of the extracranial disease and number of BM were significantly associated with survival prognosis from diagnosis of BM (Fig. 2a–d; Supplementary Table 2).

**Fig. 3:**
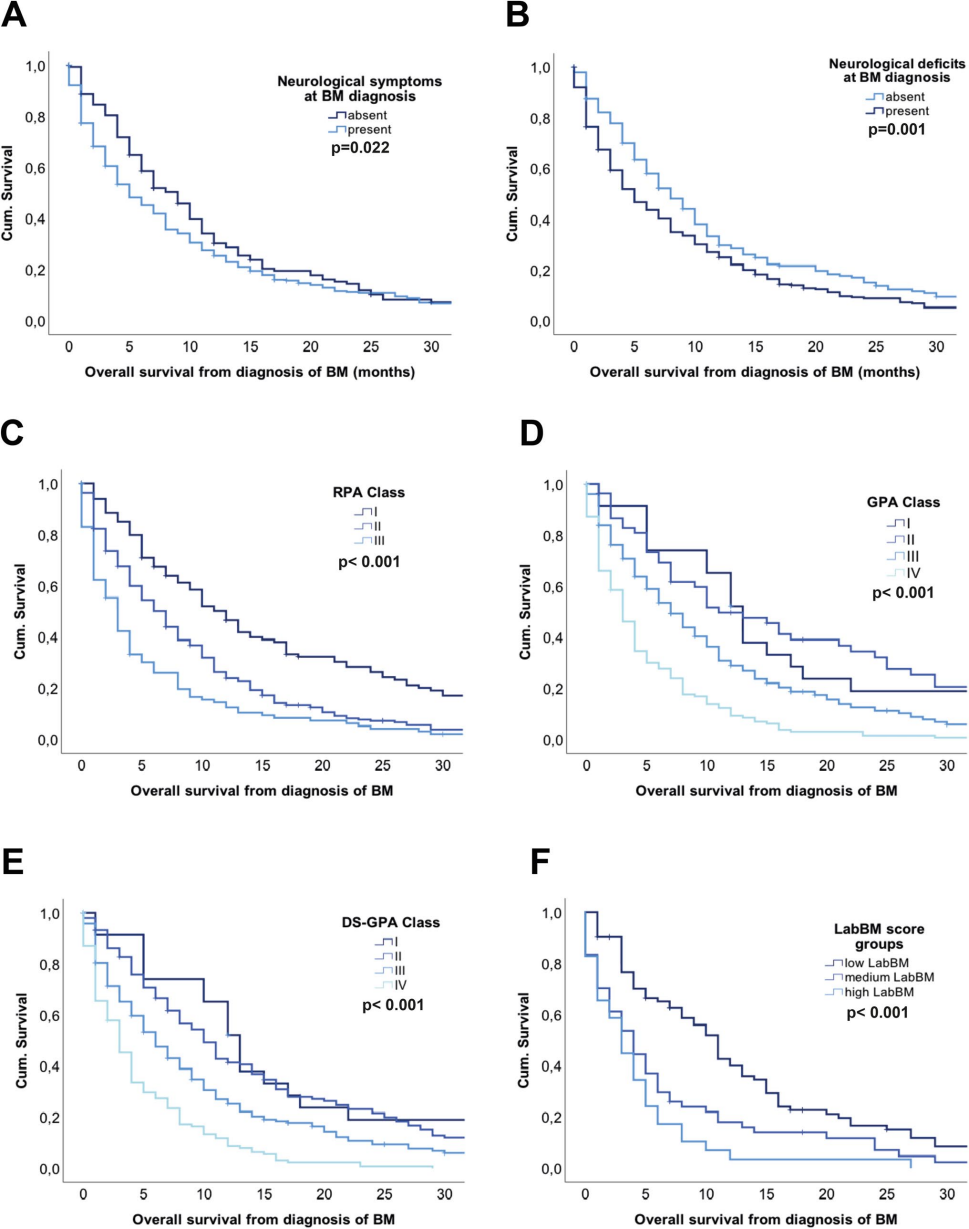
Overall survival according to (**a)** presence of neurological symptoms at BM diagnosis (**b**) presence of neurological deficits at BM diagnosis (**c**) RPA classes (**d**) GPA-classes (**e**) DS-GPA-classes (**f**) LabBM score groups

Presence of neurological symptoms was associated with impaired OS prognosis as symptomatic patients presented with a median OS of 5 months compared to 9 months in a- to oligosymptomatic patients (p = 0.030; log-rank test; Fig. 3a; Table 2). In more detail, particular presence of neurological deficits was associated with impaired OS prognosis, as patients with neurological deficits presented with a median OS of 5 months compared to 8 months in patients without neurological deficits (p = 0.001; log-rank test; Fig. 3b; Table 2). In line, signs of increased cranial pressure (6 vs. 5 months), epileptic seizures (7 vs. 5 months) and high symptomatic burden (6 vs. 5 months) were numerical associated with impaired OS prognosis, although not reaching statically significance (p > 0.05; log-rank test; Supplementary Fig. 1a–c; Supplementary Table 2; Table 2).

Time of diagnosis was not associated with a statically significant impact on OS prognosis as patients diagnosed before 2000 presented with a median overall survival of 6 months compared to 7 months in patients diagnosed and treated after 2000 (p = 0.056; log-rank test; Fig. 2e; Table 2). See Table 2 on more details of clinical characteristics impacting OS prognosis.

Survival differed between first line BM treatment approaches (p < 0.001; log-rank test; Fig. 2f; Table 2) as patients treated with neurosurgical resection showed a median OS from BM diagnosis of 11 months compared to patients who received SRS (7 months) or WBRT (5 months).

### Validation of established prognostic scores in the Vienna SCLC BM real-life cohort

The prognostic scores RPA (p < 0.001; log-rank test; Fig. 3c; Table 3), GPA (p < 0.001; log-rank test; Fig. 3d; Table 3) and DS-GPA (p < 0.001; log-rank test; Fig. 3e; Table 3) could be validated in the present real-life cohort.

**Table 3:** Survival analysis according to prognostic scores

Prognostic scores	n	Entire population (n = 489)					
		%	OS (median) in months	p-value	HR	95% CI	
RPA				< 0.001	1.66	1.44	1.91
Class I	115	23.5	12				
Class II	268	54.8	7				
Class III	106	21.7	3				
DS-GPA				< 0.001	1.60	1.43	1.80
Class I	23	4.7	13				
Class II	146	29.9	10				
Class III	188	38.4	6				
Class IV	132	27.0	3				
GPA				< 0.001	1.65	1.44	1.89
Class I	23	4.7	13				
Class II	54	11	11				
Class III	278	56.9	7				
Class IV	134	27	3				
Lab-BM				< 0.001	1.69	1.37	2.09
Low	82	49.7	11				
Medium	54	32.7	4				
High	29	17.6	3				

*CI* Confidence interval, *RPA* recursive partitioning analysis, *GPA* graded prognostic assessments, *DS-GPA* diagnostic-specific graded prognostic assessments, *HR* hazard ratio, *OS* overall survival

Complete data to calculate the LabBM score was available in 165/489 (33.7%) patients. 82/165 (49.7%) patients were categorized in low LabBM score group with a median overall survival of 11 months. The medium LabBM score group contained 54/165 (32.7%) patients showing a median survival rate of 4 months, followed by 3 months in the high LabBM score group including 29/165 (17.6%) patients (p < 0.001; log-rank test; Fig. 3f; Table 3).

### Multivariate analysis of clinical factors impacting the survival prognosis of SCLC BM patients

In multivariate analysis including DS-GPA and neurological symptoms, only DS-GPA (HR 1.58; 95% CI 1.40–1.77; p < 0.001; Cox regression model; Supplementary Table 3) remained statistically significant. In the multivariate Cox regression model of DS-GPA and neurological deficits, both, DS-GPA (HR 1.58; 95% CI 1.42–1.79; p < 0.001; Cox regression model; Supplementary Table 3) and neurological deficits (HR 1.26; 95% CI 1.04–1.53; p = 0.021; Cox regression model; Supplementary Table 3) remained statistically significant. The multivariate analysis of DS-GPA (HR 1.59; 95% CI 1.41–1.78; p < 0.001; Cox regression model; Supplementary Table 3) and synchronous diagnosis of BM (HR 0.84; 95% CI 0.69–1.02; Cox regression model; Supplementary Table 3) showed statistical significance only for DS-GPA. Furthermore, the multivariate analysis of DS-GPA and LabBM score showed statistically significant results for both variables (DS-GPA HR 1.67; 95% CI 1.36–2.05; p < 0.001; Cox regression model; LabBM score HR 1.57; CI 1.27–1.94; p < 0.001; Cox regression model; Supplementary Table 3).

## Discussion

Precise prognostic evaluation is the basis for benefit-risk adapted treatment decisions in patients with newly diagnosed SCLC BM [2, 3]. Here, we were able to analyze clinical prognostic factors as well as the recently established LabBM score, combining several standard laboratory parameters in a unique and large cohort of patients treated for SCLC BM at a single center. We could validate the application of established prognostic scores like the RPA, the GPA and the DS-GPA, as well as the LabBM score and identify presence of neurological symptoms as a prognostic parameter in patients with SCLC BM. Given the currently still detrimental survival prognosis of SCLC BM patients, clinical trials taking in account the different prognostic groups are urgently needed to formulate new treatment approaches in this patient cohort of high clinical need.

In the present real-life cohort including over 480 patients with newly diagnosed SCLC BM, the previously established prognostic scores estimating the survival based on clinical variables like the KPS, presence of extracranial disease, number of BM and age at BM diagnosis could be validated [8–10]. Importantly, the RPA, the GPA as well as the DS-GPA score were established based on clinical study cohorts, including patients enrolled in clinical trials of the Radiation Therapy Oncology Group (RTOG). The prognostic score establishment was therefore potential biased by the enrolment of a selective patient cohort eligible for clinical trials as well as the inclusion time in the late 1980s to the early 2000. The OS in our cohort treated between 1990 and 2018 was slightly longer with a median OS of 6 months, compared to 4.9 months in the cohort used to established the DS-GPA including patients treated between 1993 and 2010 [8]. Furthermore, we could validate the recently elaborated LabBM score, including standard laboratory variables as surrogate parameters for the patient’s global health status. Our findings are well in line with another smaller recent cohort postulating serum LDH concentration as an independent predictor for prognosis after WBRT for BM from SCLC [15]. Future clinical studies should therefore include the DS-GPA and potentially also the LabBM Score to identify prognostic homogenous cohorts.

Patients with neurological symptoms and in more detail with neurological deficits presented with impaired survival prognosis compared to patients with a- to oligosymptomatic disease. While the NANO scale, objectifying the symptomatic burden, is already a well-established part in the response assessment of primary brain tumors, so far the prognostic assessment in newly diagnosed BM patients did not include symptom evaluation [16]. Although screening for BM is discussed controversially in published guidelines, several centers throughout Europe frequently screen for BM in non-small cell lung cancer at diagnosis, resulting in a higher detection rate of a- or oligosymptomatic patients [17]. A further consequence of BM screening is the higher detection rate of synchronous BM at SCLC diagnosis. In the present cohort, patients with synchronous diagnosis of BM presented with a longer OS from BM diagnosis compared to patients with subsequent diagnosis. However, the OS from diagnosis of SCLC was longer in patients with subsequent BM diagnosis. Patients with synchronous diagnosis are in contrast to patients with subsequent diagnosis therapy naïve at BM diagnosis and can still receive the full repertoire including systemic therapies [13]. Indeed, BM screening potentially results in the frequent diagnosis of BM in an oligosymptomatic stage, however so far no impact of screening and earlier diagnosis of BM on OS prognosis could be validated. BM directed trials as well as BM specific endpoints in several recent trials for novel targeted and immune modulating therapies included specifically patients with oligo- to asymptomatic brain metastatic disease and could show a high intracranial, clinically meaningful efficacy for systemic treatment approaches [18–22]. In consequence, the EANO guidelines for treatment of BM patients currently suggests that systemic treatment options should be evaluated in asymptomatic BM patients suitable for a targeted therapy with proven intracranial efficacy [4]. Recently, a phase III trial of the immune checkpoint inhibitor atezolizumab in combination with chemotherapy in patients with extensive-disease SCLC, including a small number of BM patients, revealed a significantly longer overall survival and progression-free survival in the atezolizumab combination group compared to chemotherapy alone. No survival difference was observed in the particular subgroup of SCLC BM patients, however the small sample size and the missing power to answer this particular question need to be considered in the interpretation [23]. Given the high intracranial efficacy of immune checkpoint inhibitors in other entities, future clinical trials should further focus on the application of systemic therapy options in patients with SCLC BM. Here, the observed survival difference of patients with symptomatic versus patients with oligo- to asymptomatic brain metastatic disease should be considered in the clinical trial planning.

Although we were able to investigate clinical parameters in a large real-life cohort, some limitations have to be considered in the interpretation of our results. The long inclusion period from 1999 to 2018 potentially biased the results as diagnostic procedures improved over this time period. However, treatment algorithms in the particular context of SCLC BM did not change significantly. Further, the retrospective design of the study has limitation, although—to our best knowledge—this analysis is one of the so far largest real-life SCLC BM cohorts specifically addressing prognostic parameters. So far most authors concentrated on prognostic evaluation of NSCLC BM patients and structured investigation of clinical prognostic parameters in SCLC BM patients was missing [9, 24]. Here, we could include 489 patients who underwent treatment for BM from SCLC at a single institution. Therefore, the present study gives the unique opportunity to gain a deeper insight into potential prognostic factors and the clinical course of brain metastatic SCLC disease over a time period of more than 20 years.

In conclusion, this study presents a detailed clinical characterization of a unique, large real-life cohort of SCLC BM patients. Our study highlights the heterogeneity of BM treatment in a unique real-life single center cohort of SCLC BM patients and may be useful to formulate new clinical studies. Prognostic scores including the DS-GPA as well as the LabBM score could be validated for the specific cohort of SCLC BM and should be applied in future clinical trials. Further, our data stresses the importance to evaluate symptomatic and a- to oligosymptomatic patients separately due to the differing survival prognosis.

## Electronic supplementary material

Below is the link to the electronic supplementary material.

Supplementary file1—Supplementary Figure 1(PDF 1550 kb)

Supplementary file2—Supplementary Table 1 (DOCX 15 kb)

Supplementary file3—Supplementary Table 2 (DOCX 12 kb)

Supplementary file4—Supplementary Table 3 (DOCX 13 kb)

## Data Availability

The datasets generated during and/or analyzed during the current study are not publicly available due to confidentiality reasons, but anonymized versions may be available from the corresponding author on reasonable request.

## References

[1] Gavrilovic IT, Posner JB (2005). Brain metastases: epidemiology and pathophysiology. J Neurooncol.

[2] Kromer C, Xu J, Ostrom QT (2017). Estimating the annual frequency of synchronous brain metastasis in the United States 2010–2013: a population-based study. J Neurooncol.

[3] Goncalves PH, Peterson SL, Vigneau FD (2016). Risk of brain metastases in patients with nonmetastatic lung cancer: analysis of the Metropolitan Detroit Surveillance, Epidemiology, and End Results (SEER) data. Cancer.

[4] Soffietti R, Abacioglu U, Baumert B (2017). Diagnosis and treatment of brain metastases from solid tumors: guidelines from the European Association of neuro-oncology (EANO). Neuro Oncol.

[5] Bachelot T, Romieu G, Campone M (2013). Lapatinib plus capecitabine in patients with previously untreated brain metastases from HER2-positive metastatic breast cancer (LANDSCAPE): a single-group phase 2 study. Lancet Oncol.

[6] Besse B, Le Moulec S, Mazières J (2015). Bevacizumab in patients with nonsquamous non-small cell lung cancer and asymptomatic, untreated brain metastases (BRAIN): a nonrandomized, phase II study. Clin Cancer Res.

[7] Takahashi T, Yamanaka T, Seto T (2017). Prophylactic cranial irradiation versus observation in patients with extensive-disease small-cell lung cancer: a multicentre, randomised, open-label, phase 3 trial. Lancet Oncol.

[8] Sperduto PW, Kased N, Roberge D (2012). Summary report on the graded prognostic assessment: an accurate and facile diagnosis-specific tool to estimate survival for patients with brain metastases. J Clin Oncol.

[9] Sperduto CM, Watanabe Y, Mullan J (2008). A validation study of a new prognostic index for patients with brain metastases: the Graded Prognostic Assessment. J Neurosurg.

[10] Gaspar L, Scott C, Rotman M (1997). Recursive partitioning analysis (RPA) of prognostic factors in three Radiation Therapy Oncology Group (RTOG) brain metastases trials. Int J Radiat Oncol Biol Phys.

[11] Berghoff AS, Wolpert F, Holland-Letz T (2017). Combining standard clinical blood values for improving survival prediction in patients with newly diagnosed brain metastases-development and validation of the LabBM score. Neuro Oncol.

[12] Berghoff AS, Schur S, Füreder LM (2016). Descriptive statistical analysis of a real life cohort of 2419 patients with brain metastases of solid cancers. ESMO Open.

[13] Früh M, De Ruysscher D, Popat S (2013). Small-cell lung cancer (SCLC): ESMO clinical practice guidelines for diagnosis, treatment and follow-up. Ann Oncol.

[14] Bender R, Lange S (2001). Adjusting for multiple testing—when and how?. J Clin Epidemiol.

[15] Anami S, Doi H, Nakamatsu K (2018). Serum lactate dehydrogenase predicts survival in small-cell lung cancer patients with brain metastases that were treated with whole-brain radiotherapy. J Radiat Res.

[16] Nayak L, Deangelis LM, Brandes AA (2017). The Neurologic Assessment in Neuro-Oncology (NANO) scale: a tool to assess neurologic function for integration into the response assessment in neuro-oncology (RANO) criteria. Neuro Oncol.

[17] Kim SY, Kim JS, Park HS (2005). Screening of brain metastasis with limited magnetic resonance imaging (MRI): clinical implications of using limited brain MRI during initial staging for non-small cell lung cancer patients. J Korean Med Sci.

[18] Shaw AT, Peters S, Mok T (2017). Alectinib versus crizotinib in treatment-naive advanced *ALK* -positive non-small cell lung cancer (NSCLC): primary results of the global phase III ALEX study. J Clin Oncol.

[19] Goldberg SB, Gettinger SN, Mahajan A (2016). Pembrolizumab for patients with melanoma or non-small-cell lung cancer and untreated brain metastases: early analysis of a non-randomised, open-label, phase 2 trial. Lancet Oncol.

[20] Israel B, Margolin K, Ernstoff MS (2012). Ipilimumab in patients with melanoma and brain metastases: an open-label, phase 2 trial. Lancet Oncol.

[21] Novello S, Mazieres J, Oh I-J (2017). Primary results from the phase III ALUR study of alectinib versus chemotherapy in previously treated ALK+ non-small-cell lung cancer (NSCLC). Ann Oncol.

[22] Davies MA, Saiag P, Robert C (2017). Dabrafenib plus trametinib in patients with BRAFV600-mutant melanoma brain metastases (COMBI-MB): a multicentre, multicohort, open-label, phase 2 trial. Lancet Oncol.

[23] Horn L, Mansfield AS, Szczęsna A (2018). First-line atezolizumab plus chemotherapy in extensive-stage small-cell lung cancer. N Engl J Med.

[24] Berghoff AS, Schur S, Fuereder L (2016). Descriptive analysis of 2419 patients with brain metastases of solid cancers: a real life cohort. J Clin Oncol.

