# Supplementary figures and images for: Prognostic assessment in patients with newly diagnosed small cell lung cancer brain metastases: results from a real-life cohort
Source: J Neurooncol. 2019 Aug 27;145(1):85–95. doi: 10.1007/s11060-019-03269-x (PMC6775039; doi:10.1007/s11060-019-03269-x)

# SUPPLEMENTARY FIGURES

A

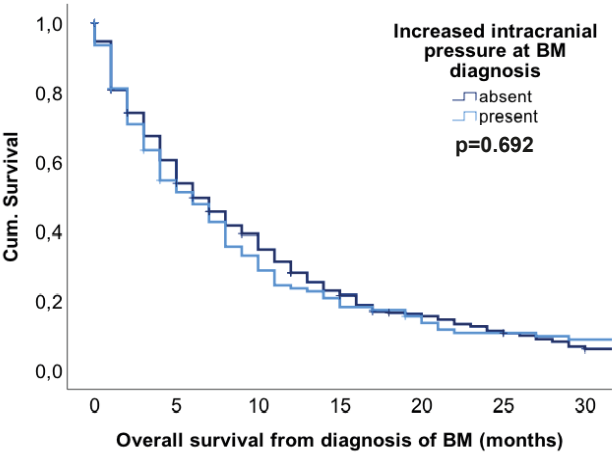

B

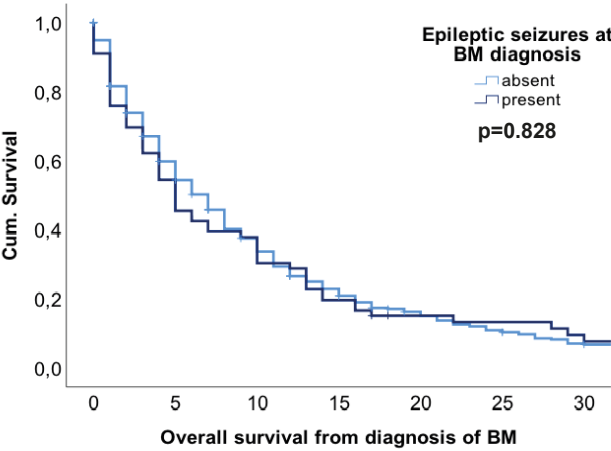

C

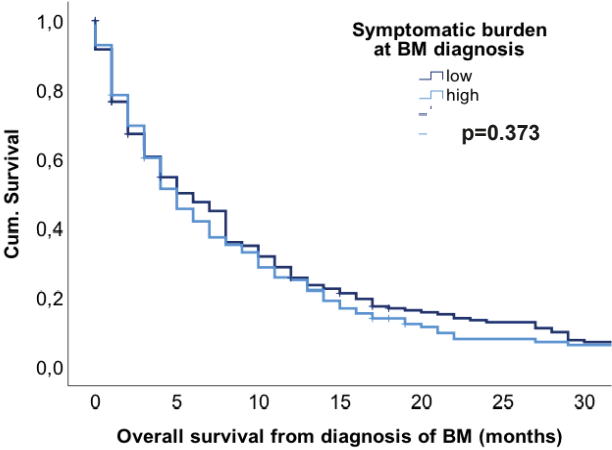

Supplement: Supplementary file 1 — Supplementary file1—Supplementary Figure 1(PDF 1550 kb) [file 11060_2019_3269_MOESM1_ESM.pdf]
