# Supplementary material for: Prognostic assessment in patients with newly diagnosed small cell lung cancer brain metastases: results from a real-life cohort
Source: J Neurooncol. 2019 Aug 27;145(1):85–95. doi: 10.1007/s11060-019-03269-x (PMC6775039; doi:10.1007/s11060-019-03269-x)
Supplement: Supplementary file 2 — Supplementary file2—Supplementary Table 1 (DOCX 15 kb) [file 11060_2019_3269_MOESM2_ESM.docx]

**Supplementary Table 1:** Further patient’s and clinical characteristics

| **Characteristics of total patient’s cohort** | | **Entire Population**  **(n= 489)** | |
| --- | --- | --- | --- |
|  | | **n** | **%** |
| Median age at SCLC diagnosis | | 61 (38-88) | |
| Stage IV at diagnosis of SCLC | |  |  |
| Yes | | 323 | 66.1 |
| No | | 166 | 33.9 |
| Disease status at diagnosis of SCLC | |  |  |
| Limited disease | | 158 | 32.3 |
| Extensive disease | | 331 | 67.7 |
| Extracranial metastases | |  |  |
| Present | | 266 | 54.4 |
| Visceral | | 173 | 35.4 |
| Lung | | 59 | 12.1 |
| Hepatic | | 81 | 16.6 |
| Absent | | 223 | 45.6 |
| PCI before diagnosis of BM | |  |  |
| Yes | | 62 | 12.6 |
| No | | 427 | 87.3 |
| **Characteristics at BM diagnosis** | | | |
| Year of diagnosis |  | |  |
| ≤ 2000 | 178 | | 36.5% |
| > 2000 | 311 | | 63.5% |
| Localization of BM |  | |  |
| Supratentorial | 260 | | 53.2% |
| Infratentorial | 66 | | 13.5% |
| Both | 163 | | 33.3% |
| Brainstem BM |  | |  |
| Yes | 27 | | 5.5% |
| No | 462 | | 94.5% |
| Leptomeningeal encolvement |  | |  |
| Yes | 4 | | 0.08% |
| No | 485 | | 99.2% |
| Number of BM |  | |  |
| 1 | 203 | | 41.5% |
| 2 to 3 | 138 | | 28.2% |
| >=4 | 148 | | 30.3% |
| Size of BM at diagnosis |  | |  |
| <3cm | 354 | | 72.4% |
| >=3cm | 135 | | 27.6% |
| **Characteristics after BM diagnosis** | | | |
| SRS |  | |  |
| to singular BM | 106 | | 21.7% |
| to 2-3 BM | 79 | | 16.2% |
| to ≥ 4 BM | 27 | | 5.5% |
| to symptomatic BM | 132 | | 26.9% |
| WBRT |  | |  |
| to singular BM | 39 | | 8.0% |
| to 2-3 BM | 35 | | 7.2% |
| to ≥ 4 BM | 27 | | 5.5% |
| to symptomatic BM | 123 | | 25.2% |
| Neurosurgical resection |  | |  |
| to singular BM | 49 | | 10.0% |
| to 2-3 BM | 21 | | 4.3% |
| to ≥ 4 BM | 5 | | 1.0% |
| to symptomatic BM | 69 | | 14.1% |
| BM biopsy (without complete resection) | 5 | | 6.7% |
| Complete resections | 70 | | 93.3% |
| Brain only metastatic disease during the entire course of disease |  | |  |
| Yes | 140 | | 28.6% |
| No | 349 | | 71.4% |
| Cause of death |  | |  |
| Intracranial progression | 19 | | 3.9% |
| Systemic progression | 133 | | 27.2% |
| Intracranial and systemic progression | 176 | | 36.0% |
| other reasons (not cancer associated) | 11 | | 2.2% |
| Myocardial infarction | 5 | | 1.0% |
| Severe perioperative complications | 1 | | 0.2% |
| Sepsis | 4 | | 0.8% |
| Traumatic subarachnoid hemorrhage | 1 | | 0.2% |
| No data on cause of death available | 150 | | 30.7% |

Abbreviations: BM: Brain metastases, PCI: Prophylactic cranial irradiation, SCLC: small cell lung cancer, SRS: Stereotactic radiosurgery, WBRT: whole brain radiation therapy
