# Supplementary material for: Prognostic assessment in patients with newly diagnosed small cell lung cancer brain metastases: results from a real-life cohort
Source: J Neurooncol. 2019 Aug 27;145(1):85–95. doi: 10.1007/s11060-019-03269-x (PMC6775039; doi:10.1007/s11060-019-03269-x)
Supplement: Supplementary file 3 — Supplementary file3—Supplementary Table 2 (DOCX 12 kb) [file 11060_2019_3269_MOESM3_ESM.docx]

**Supplementary Table 2:** Overall survival according prognostic factors included in DS-GPA

| **Characteristics** | **Entire Population (n= 489)** | |
| --- | --- | --- |
|  |  |  |
|  | **OS (median)**  **in months** | **p-value** |
| Age at BM diagnosis |  | *< 0.001* |
| <50 | 10 |  |
| 50-60 | 7 |  |
| >60 | 5 |  |
| KPS at BM diagnosis |  | *< 0.001* |
| < 70 | 3 |  |
| 70-80 | 7 |  |
| 90-100 | 10 |  |
| Number of BM |  | *< 0.001* |
| 1 | 8 |  |
| 2 to 3 | 7 |  |
| >=4 | 4 |  |
| Extracranial metastases |  | *< 0.001* |
| Present | 3 |  |
| Absent | 9 |  |

Abbreviations: BM: Brain Metastases, KPS: Karnofsky Performance Score
