# Supplementary material for: Prognostic assessment in patients with newly diagnosed small cell lung cancer brain metastases: results from a real-life cohort
Source: J Neurooncol. 2019 Aug 27;145(1):85–95. doi: 10.1007/s11060-019-03269-x (PMC6775039; doi:10.1007/s11060-019-03269-x)
Supplement: Supplementary file 4 — Supplementary file4—Supplementary Table 3 (DOCX 13 kb) [file 11060_2019_3269_MOESM4_ESM.docx]

**Supplementary Table 3:** Results of multivariate testing of clinical prognostic factor

|  | **Hazard ratio** | **p-value** | **Confidence interval** | |
| --- | --- | --- | --- | --- |
| DS-GPA | 1.58 | <0.001 | 1.40 | 1.77 |
| Neurological deficits | 1.26 | 0.021 | 1.04 | 1.53 |
|  |  |  |  |  |
| DS-GPA | 1.59 | <0.001 | 1.42 | 1.79 |
| Neurological symptoms | 1.17 | 0.135 | 0.95 | 1.44 |
|  |  |  |  |  |
| DS-GPA | 1.59 | <0.001 | 1.41 | 1.78 |
| Synchronous diagnosis of BM | 0.84 | 0.072 | 0.69 | 1.02 |
|  |  |  |  |  |
| DS-GPA | 1.67 | <0.001 | 1.36 | 2.05 |
| LabBM score | 1.57 | <0.001 | 1.27 | 1.94 |

Abbreviations: BM: Brain Metastases; DS-GPA: Diagnostic-Specific Graded Prognostic Assessments
